# Temperature-Associated SD Card Logging Failures in PurpleAir Monitors: Findings from the Strong Heart Air Study

**DOI:** 10.3390/s26216923

**Published:** 2026-11-01

**Authors:** Max Ulibarri, James Ross, Whitney Collado, Anna Tillery, Jason Umans, Mary Mohr, Samantha Ishak, Nicole Amodio, Tauqeer Ali, Laura Smith, Rae O’Leary, McKinzie Circle Eagle, Rebecca White Bull, Ana Navas-Acien, Steven Chillrud, Markus Hilpert

**Affiliations:** 1 Vagelos College of Physicians and Surgeons, Columbia University Irving Medical Center, New York, NY 10032, USA; 2 Lamont-Doherty Earth Observatory, Columbia University, New York, NY 10027, USA; 3 Department of Environmental Health Sciences, Mailman School of Public Health, Columbia University Irving Medical Center, New York, NY 10032, USA; 4 MedStar Health Research Institute, Hyattsville, MD 20782, USA; 5 Center for American Indian Health Research, Hudson College of Public Health, University of Oklahoma Health Sciences Center, Oklahoma City, OK 73104, USA; 6 Missouri Breaks Industries Research Incorporation, Eagle Butte, SD 57625, USA

**Keywords:** low-cost sensors, PurpleAir, PM_2.5_, sensor performance, temperature effects, field deployment

## Abstract

**Highlights:**

**What are the main findings?**

- A popular type of low-cost air quality monitors using local SD card storage experience substantial data loss at low temperatures despite operating above the manufacturer-reported lower temperature limit.
- Field experiments demonstrated that SD card logging became unreliable at approximately 4–8 °C when WiFi connectivity was absent.

**What are the implications of the main findings?**

- Studies using low-cost PM_2.5_ monitors in cold climates should evaluate temperature-related data loss during study design and pilot testing.
- Local SD card storage can introduce an important data completeness limitation when cloud-based data transmission is not used.

**Abstract:**

Low-cost air quality monitors are increasingly used in environmental health studies. We report the deployment and evaluation of low-cost, fine particulate matter (PM_2.5_) monitors in an ongoing investigation of air quality and cardiovascular health in three climatologically distinct American Indian communities. Prior to deploying large numbers of PurpleAir monitors for this work, we conducted pilot collocations of ten monitors at each study site to evaluate performance under field conditions. Monitors were configured to store data locally on SD cards rather than upload via WiFi to avoid cloud-based data ownership concerns in partner American Indian communities. During pilot collocations, we observed substantial data loss in colder regions: data recovery was 57% in Oklahoma and 10% in South Dakota, compared with 100% in Arizona. SD card logging commonly failed when ambient temperatures fell to mean (standard deviation) levels of 4.4 (3.2) °C in Oklahoma and 7.7 (2.0) °C in South Dakota. Follow-up outdoor experiments suggested that WiFi connection improves SD card reliability at low temperatures, while controlled 4 °C cold-room experiments confirmed logging failures without WiFi. Our findings demonstrate temperature-related limitations of PurpleAir monitors when used without WiFi and provide practical guidance for low-cost PM_2.5_ monitoring in cold climates.

## 1. Introduction

Low-cost air quality monitors are widely used by citizen scientists, municipalities, environmental groups, and increasingly in academic research [1,2,3]. A commonly used monitor is the PurpleAir monitor, whose performance has been extensively evaluated by the scientific community [4,5]. Further, the US EPA’s online “AirNow” Fire and Smoke map is based on the readings of nationwide outdoor PurpleAir monitors, whose data have been made publicly available by the numerous operators of the monitors, which use WiFi connection to upload measured data automatically to the PurpleAir cloud [6,7].

PurpleAir monitors are popular for multiple reasons, including cost, and the use of dual PM sensors, providing the ability for quality control checks and flexible data storage options. Data measured on these devices can either be transmitted to the cloud via a WiFi network connection and/or stored locally on microSD cards that can be inserted into the monitors’ card slots. Local SD card storage allows for data collection without WiFi or cloud-based data transmissions, which can be important in settings where no WiFi is available or where local control of data storage is required. The SD cards are not required when data are stored in the cloud and must have a minimum write speed of 10 megabytes per second and storage capacity of up to 32 GB. Like many low-cost sensors, PurpleAir monitors record an internal temperature and relative humidity that are impacted by multiple factors, including heat from operation and charging, and solar heating, which varies by time of day, windspeed, and cloud cover. As such, the internal temperature is higher than true ambient temperature.

Due to the widespread use of PurpleAir monitors and their ability to be calibrated through collocation with reference instruments prior to field deployment, we elected to use PurpleAir monitors in a study aimed to examine the short-term effects of PM_2.5_ air pollution on ambulatory blood pressure levels and long-term impacts on incident cardiovascular disease [8]. This study is conducted in American Indian communities located in three states (Arizona, Oklahoma and South Dakota) that are climatologically distinct. The technical specifications of the particulate matter sensors used in the PurpleAir monitors, and of the PurpleAir monitors themselves, report an operating temperature range from −10 °C to 60 °C, and our plan was not to deploy the monitors if temperature during a field deployment was predicted to fall outside this range [9,10]. PurpleAir models with SD cards are often chosen when WiFi is not available or to avoid data ownership issues since the PurpleAir company owns data uploaded to their cloud server. To not violate tribal principles of data sovereignty [11,12], we used only SD cards to record PurpleAir data.

The purpose of this note is to report the results of the air monitor collocations, including gaps in measured air quality data unexpectedly encountered at temperatures well above the reported minimum operating temperature of −10 °C. We report the fieldwork collocations results side by side with the results of temperature-controlled experiments that we conducted to confirm the findings of temperature-driven data gaps in the collocations.

## 2. Material & Methods

PurpleAir Flex monitors (PurpleAir, LLC, Bluffdale, UT, USA) were purchased in 2024 and used in a pilot collocation test in the office of the corresponding author, in an indoor New York City environment at room temperature. The collocation data were recorded on SD cards inserted into the monitors (SanDisk Corporation, Milpitas, CA, USA, 32 GB Ultra microSD) rather than uploaded to the cloud storage, aligned with the data storage approach that would be used during monitor deployment in study participant homes. These SD cards had a minimum operating temperature of −25 °C [13]. The PurpleAir units were powered using standard US household electrical outlets. All PurpleAir monitors used firmware version 7.04 throughout this study, including this initial indoor collocation and all subsequent field and laboratory experiments. The multi-day collocation test conducted indoors went well, and no issues or data gaps on the SD cards were identified, though the monitors remained at room temperature for the duration of the experiment.

Following this successful indoor collocation, ten monitors were collocated outdoors at each of the three study sites in Eagle Butte, South Dakota; Lawton, Oklahoma; and Scottsdale, Arizona. The deployment periods and corresponding ambient temperature distributions are provided in Table 1. After substantial data gaps were found in the South Dakota data, additional experiments were conducted in New York City to reproduce the data losses and identify their cause. A monitor connected to WiFi was placed outdoors on a rooftop of an apartment building. Despite a low outdoor temperature around −1 °C, similar to South Dakota, this monitor did not reproduce the significant data gaps on its SD card. PurpleAir technical support staff, from whom we sought technical assistance, suggested that SD card logging would be more reliable when also transmitting data via WiFi. An additional 7-day experiment (22 February 2025 to 1 March 2025) was conducted outdoors in New York City where two monitors with SD cards were collocated, one with and the other without WiFi connection; ambient temperatures in this experiment ranged between 1.1–13.9 °C. Finally, two monitors that were not connected to WiFi were placed for three days (21 March 2025 to 24 March 2025) in a 4 °C walk-in refrigerator to evaluate SD card logging in a constant low temperature environment. In the NYC experiments, the monitors were powered by 5V DC, 2.5A switching power supplies connected to standard US electrical outlets (120 V AC), whereas for the collocations at the three study sites, a 25-port USB charger station was used. All reported experiments used the same SanDisk Ultra 32 GB microSD card model. Additional tests were conducted in the 4 °C walk-in refrigerator using an alternative brand of SD cards (ADATA Technology Co., Ltd., New Taipei City, Taiwan, Premier microSDHC UHS-I Class 10 32 GB) to assess whether card selection affected logging reliability. These tests did not demonstrate improved logging performance at low temperatures, and the data are not presented here.

To explore potential associations between SD card data recovery and ambient temperature at each study site, we determined Pearson’s correlation coefficients between hourly data recovery and temperature. To account for temporal autocorrelation, 95% confidence intervals (CIs) were calculated using heteroskedasticity- and autocorrelation-consistent (HAC) standard errors with a Bartlett kernel and a maximum lag of 48 h. We defined hourly data recovery as the actual number of data points logged by the 10 monitors per hour divided by 300 (reflecting perfect data recovery with measurements every 2 min for all 10 monitors combined) and multiplied by 100%. Hourly temperature data for the three monitor collocations were obtained for the closest weather station from the National Oceanic and Atmospheric Administration (NOAA) Integrated Surface Dataset. The stations were located approximately 63 km from the South Dakota site, 5 km from the Oklahoma site, and 11 km from the Arizona site.

To determine the conditions for which SD card data collection became unreliable at each study site, we identified the points in time when data recovery became lower than 90%, an operational threshold selected to indicate meaningful data loss relative to the expected complete data record. A new failure event was identified each time data recovery dropped below the threshold following a period of resumed data recording. For these points in time, we obtained ambient temperatures (summarized by their mean and standard deviation) from the nearest NOAA weather station and assumed it to be the failure temperature *T_off_* at which SD card logging ceased. From the temperature data, we also determined the sign of the time derivative of temperature, sgn(*dT*/*dt*), where sgn is the signum function. To explore potential short-duration data losses, we also conducted the analyses at a 10 min resolution. The corresponding 10 min temperatures were estimated from the hourly records through linear interpolation.

## 3. Results

### 3.1. Collocations

Table 1 summarizes the collocations at the three study sites, while Supplementary Figure S1 shows the hourly time series of data recovery and hourly ambient temperature. Overall, data recovery ($\bar{I}$) was lowest in South Dakota (9.4%), followed by Oklahoma (56.6%) and Arizona (100%), where no losses occurred. Device-level data recovery was relatively consistent within each site, ranging from 50.4% to 59.9% in Oklahoma and 6.1% to 13.9% in South Dakota. All monitors in Arizona achieved 100% data recovery. Hourly data recovery was positively correlated with ambient air temperature in Oklahoma (*r* = 0.79, *n* = 1642, 95% CI: 0.74–0.82) and South Dakota (*r* = 0.54, *n* = 982, 95% CI: 0.45–0.63). Sensitivity analyses using HAC maximum lags of 24, 72, and 168 h yielded similar confidence intervals at both sites.

At the Oklahoma site, hourly data recovery dropped below 90% at a mean failure temperature *T_off_* = 4.4 °C, and at the South Dakota site at a mean *T_off_* = 7.7 °C (Supplementary Figure S1). In Oklahoma, the temperature as recorded by NOAA weather stations was decreasing at 89% of the time when data recording failed; increasing at 9% of the time; and remaining stable at 2% of the time. In South Dakota, the temperature was decreasing at 75% of the time and increasing at 25% of the time when data recording failed. Ambient temperature exceeded the site-specific mean failure temperature *T_off_* during 55.7% of hourly observations in Oklahoma and 6.8% in South Dakota, closely corresponding to the respective overall data recovery rates in each region. The mean internal temperature of the PurpleAir devices when data recording ceased, as recorded by the last valid data point from the monitors themselves (*T_i_*), was higher than the corresponding ambient temperatures (13.8 °C in South Dakota and 10.6 °C in Oklahoma).

Sensitivity analyses using 80% and 95% recovery thresholds yielded similar results, with mean failure temperatures of 4.2 °C and 4.9 °C in Oklahoma and 7.2 °C and 8.1 °C in South Dakota, respectively, at these thresholds. The number of identified failures at 80% and 95% recovery thresholds were 44 and 47 in Oklahoma and 14 and 12 in South Dakota, respectively.

### 3.2. Effects of WiFi Connection

**Figure 1:**
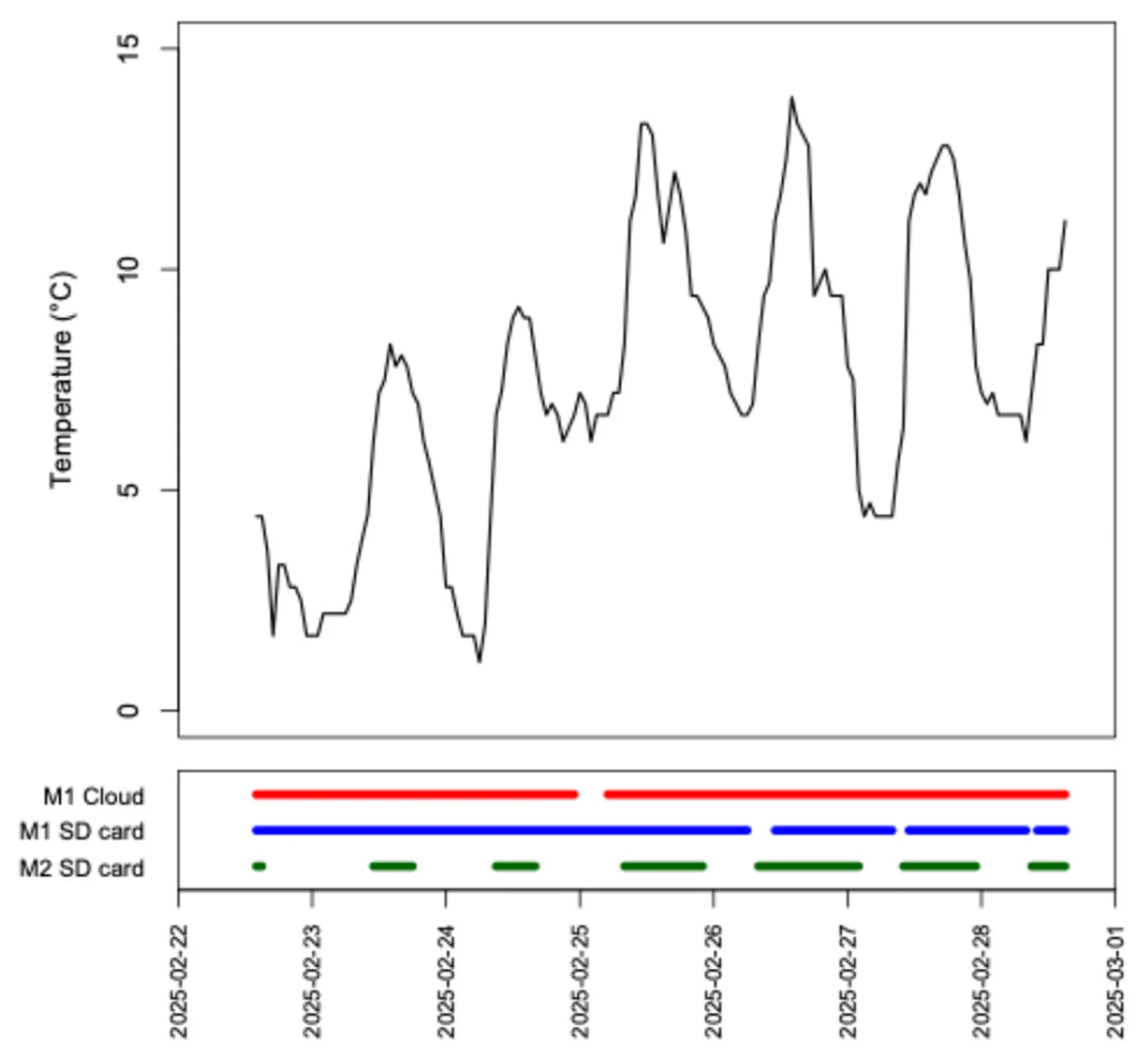
Comparison of two PurpleAir monitors collocated outdoors on a New York City rooftop. Both are equipped with a memory SD card, but Monitor 1 (M1) is connected to WiFi, while Monitor 2 (M2) is not. The upper panel shows ambient temperature, while the lower panel shows data-logging status. Colored lines indicate periods of successful data storage to the corresponding medium, while gaps indicate periods when no data were recorded.

Figure 1 shows the results of the 7-day New York City experiment, which sought to elucidate the potential impact of the WiFi connection of the air quality monitor on SD card logging. Monitor 2, the monitor without WiFi connection, had markedly bigger data gaps on its SD card than Monitor 1, the monitor with WiFi connection. Overall SD card data recovery was 93% for Monitor 1 and 45% for Monitor 2. Similar to the three study sites, data recovery ceased when temperatures began to decrease, at a mean (standard deviation) *T_off_* = 6.7 (1.7) °C, with a mean (standard deviation) *T_i_* = 11.4 (0.7) °C for Monitor 2. Monitor 1 lost WiFi connectivity for several hours on 25 February 2025. However, during this period, the measured data were still successfully logged to the SD card.

At a 10-min temporal resolution, we identified 4 additional brief data gaps (Supplementary Figure S2). These gaps occurred at a mean temperature of 8.9 °C within 10–35 min after the monitors rewarmed above *T_off_* and resumed writing data to the SD card.

### 3.3. Tests Under Controlled Thermal Conditions

Additional experiments using two monitors without WiFi connection were conducted in a controlled thermal environment, a walk-in 4 °C refrigerator. After placing the monitors in the refrigerator, we periodically checked whether the blue LED on the monitors would light up every two minutes, indicating that, according to the manufacturer, the measured data were uploaded to the SD card. Indeed, the blue LED lit up every two minutes. However, when we stopped the experiment after three days, we found that the monitors stopped logging data to the SD card about 80 min after they were put into the refrigerator (Supplementary Figure S3), suggesting that the LED indicator does not guarantee successful SD card writing. Data logging stopped when the air monitors dropped to an internal temperature (*T_i_*) of 12 °C, similar to *T_i_* found in our prior experiments. This temperature is substantially higher than 4 °C, the actual temperature in the refrigerator, and significantly higher than the manufacturer-reported −10 °C lower operating limit.

## 4. Conclusions

The PurpleAir Flex Monitors unexpectedly failed to log measured data to an SD card at colder ambient temperatures, with data loss occurring on average at 4.4 °C in Oklahoma and 7.7 °C in South Dakota in our field experiments. These are temperatures well above the manufacturer’s reported lower operating limit of −10 °C, which applies to the device and sensor together and is not specific to WiFi connectivity. These data failures are not likely explained by the SD cards themselves, given that the two brands of SD cards we used (SanDisk and ADATA) had a lower operating limit of −25 °C. Our experiments indicate that connecting the monitor to a WiFi network markedly improves data recovery at low temperatures under our tested conditions. The mechanism is not entirely clear, although the manufacturer (private communication) suggested that a lack of WiFi connection might affect the internal temperature of the monitor, because, without WiFi, the monitor reportedly reboots every 20 min.

After we identified the problem in January 2025, two other research groups reported similar SD card failures at moderately low temperatures around 10 °C on a PurpleAir forum [14]. After an investigation, the air quality manufacturer acknowledged the flaw and was able to reproduce the error. These observations suggest that low-cost PurpleAir monitors, despite their widespread use in citizen-science projects, may be vulnerable to temperature-dependent data loss when used without WiFi.

The limitations of our study include assessing the impact of the WiFi connection using two monitors (Figure 1) without a crossover test where the WiFi connection is exchanged between the two monitors, which would reduce any inter-device variability, in addition to reliance on temperature measurements from the nearest NOAA weather station rather than measurements taken immediately adjacent to the monitors. Spatial differences in temperature, particularly in South Dakota where the reference station was approximately 63 km away, may have introduced uncertainty into the estimated temperatures at which SD card logging failed. This may partly explain why SD card logging occasionally ceased in South Dakota when temperatures were rising, although factors such as solar radiation or wind chill may also contribute. Given these challenges, we incorporated the Atmocube (Atmotech, Inc., San Francisco, CA, USA) in place of the PurpleAir in South Dakota because exploratory testing suggested greater cold hardiness. In Oklahoma, where PurpleAir performance was somewhat better though still variable, we plan to deploy the Atmocube monitors, particularly during the colder winter months.

Overall, these findings demonstrate that while low-cost air quality monitors remain valuable tools for public health studies, temperature conditions and WiFi connectivity appear to have important implications for data reliability when local SD card storage is used. Researchers planning field deployments in colder climates or in settings where WiFi cannot be relied upon may wish to evaluate sensor performance under anticipated environmental conditions and consider alternative devices or modified deployment protocols.

## Figures and Tables

**Table 1:** Summary of monitoring conditions and data recovery at the three study sites.

	South Dakota	Oklahoma	Arizona
Period	13 November 2024–29 December 2024	21 November 2024–29 January 2025	9 April 2025–17 April 2025
Weather station	Faith Municipal Airport, SD	Lawton Municipal Airport, OK	Phoenix airport, AZ
Mean (standard deviation) temperature *T*	−3.0 (6.9) °C	5.7 (6.9) °C	28.6 (5.2) °C
Median (25th, 75th) temperature *T*	−2.8 (−7.8, 1.7) °C	6.1 (0.0, 10.6) °C	28.3 (23.7, 32.8) °C
Number of *T_off_*	12	46	0
Mean (standard deviation) *T_off_*	7.7 (2.0) °C	4.4 (3.2) °C	N/A
Mean (standard deviation) *T_i_*	13.8 (4.1) °C	10.6 (0.7) °C	N/A
*dT*/*dt* < 0 @ *T_off_*	75%	89%	N/A
Mean data recovery $\bar{I}$	9.4%	56.6%	100%
% T > mean *T_off_*	6.8%	55.7%	N/A
Pearson’s *r_I_*_,*T*_ (95% CI)	0.54 (0.45–0.63)	0.79 (0.74–0.82)	N/A

## Data Availability

The original contributions presented in this study are included in the article/Supplementary Materials. Further inquiries can be directed to the corresponding author(s).

## Supplementary Materials

The following supporting information can be downloaded at: https://www.mdpi.com/article/10.3390/s26216923/s1. Supplemental Figure S1: Hourly times series of percent data recovery and ambient temperature for the three study sites. Circles and triangles indicate the points in time when data recovery dropped below 90%, with temperature measured at the nearest weather station either decreasing or increasing, respectively. Supplemental Figure S2: Comparison of two Purple Air monitors collocated outdoors on a rooftop at a ten-minute resolution. Both monitors were equipped with SD cards but only Monitor 1 (M1) was connected to WiFi, while Monitor 2 (M2) was not. The upper panel shows ambient temperature, while the lower panel shows data logging status. Colored lines indicate periods of successful data storage to the corresponding medium, while gaps indicate periods when no data were recorded. Green vertical ticks are instances of failed data recordings. Supplemental Figure S3: Time series of temperature (internal) and PM2.5 levels logged by the SD cards of two air monitors (#1 and #2) that were placed at zero time into a 4 °C walk-in refrigerator. The time series end at about 80 min when both monitors stopped logging data to the SD card. PM2.5 levels quickly approach 0 μg/m^3^ in the refrigerator due to lack of air exchange.
