# Supplementary material for: Temperature-Associated SD Card Logging Failures in PurpleAir Monitors: Findings from the Strong Heart Air Study
Source: Sensors (Basel). 2026 Nov 1;26(21):6923. doi: 10.3390/s26216923 (PMC13766475; doi:10.3390/s26216923)
Supplement: Supplementary file 1 [file sensors-26-06923-s001.zip › Supplemental Figure S1.pdf]

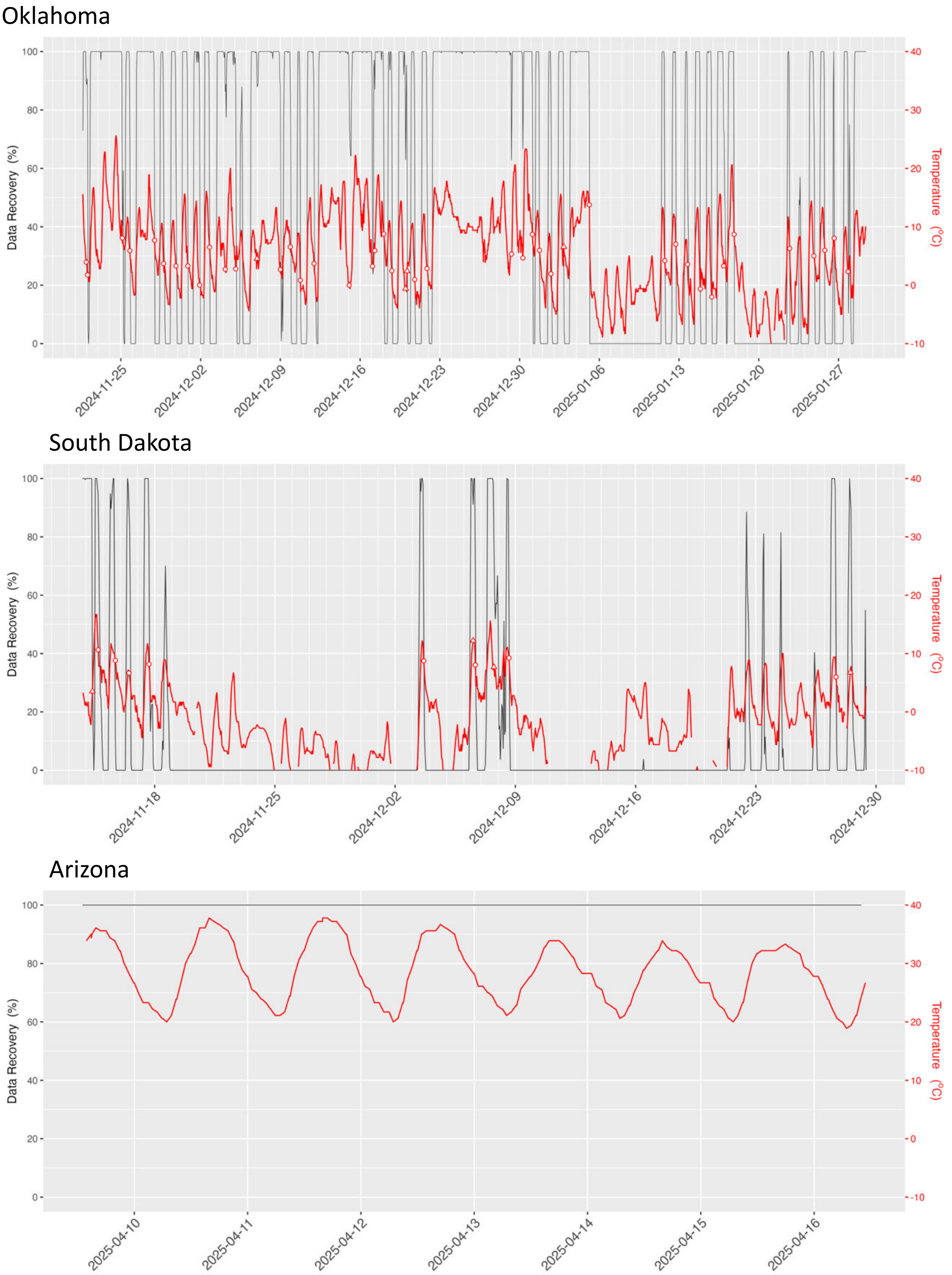

Supplemental Figure S1: Hourly times series of percent data recovery and ambient temperature for the three study sites. Circles and triangles indicate the points in time when data recovery dropped below 90%, with temperature measured at the nearest weather station either decreasing or increasing, respectively.
