# Supplementary material for: Temperature-Associated SD Card Logging Failures in PurpleAir Monitors: Findings from the Strong Heart Air Study
Source: Sensors (Basel). 2026 Nov 1;26(21):6923. doi: 10.3390/s26216923 (PMC13766475; doi:10.3390/s26216923)
Supplement: Supplementary file 1 [file sensors-26-06923-s001.zip › Supplemental Figure S2.pdf]

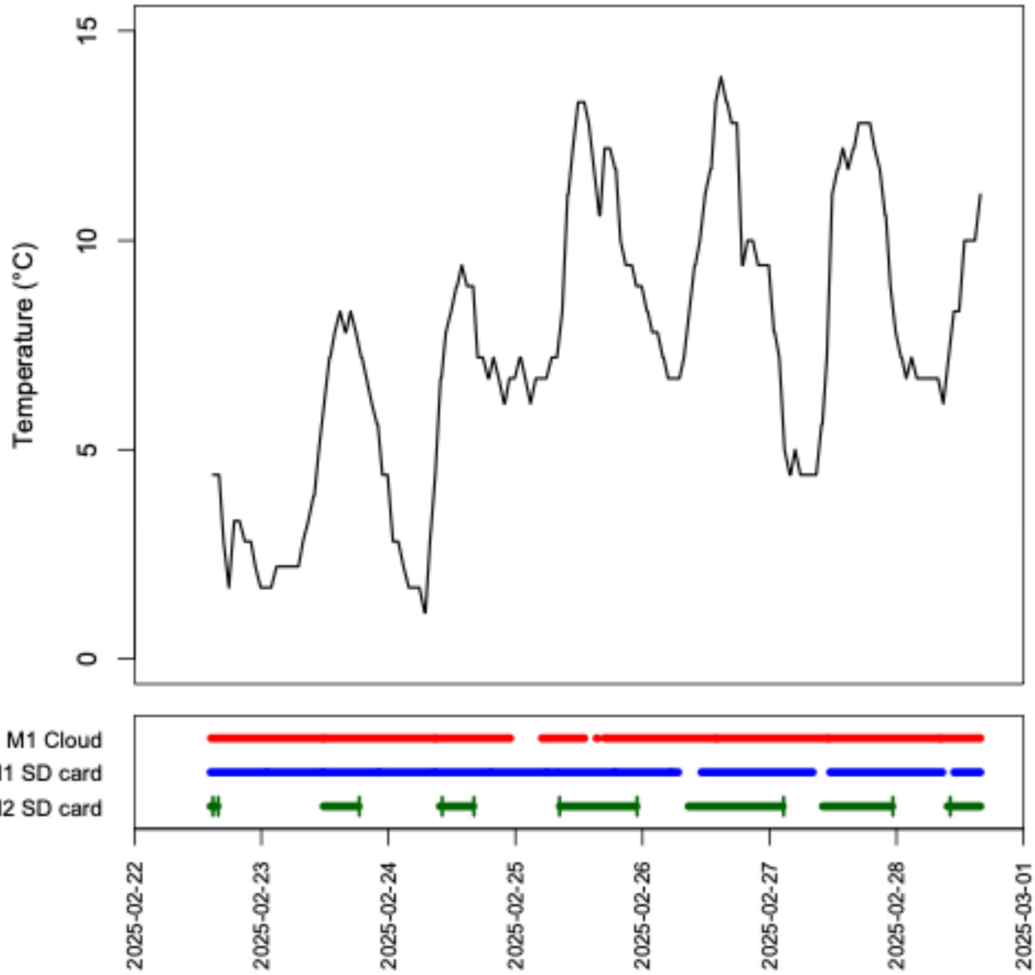

Supplemental Figure S2: Comparison of two Purple Air monitors collocated outdoors on a rooftop at a ten-minute resolution. Both monitors were equipped with SD cards but only Monitor 1 (M1) was connected to WiFi, while Monitor 2 (M2) was not. The upper panel shows ambient temperature, while the lower panel shows data logging status. Colored lines indicate periods of successful data storage to the corresponding medium, while gaps indicate periods when no data were recorded. Green vertical ticks are instances of failed data recordings.
