# Supplementary material for: Temperature-Associated SD Card Logging Failures in PurpleAir Monitors: Findings from the Strong Heart Air Study
Source: Sensors (Basel). 2026 Nov 1;26(21):6923. doi: 10.3390/s26216923 (PMC13766475; doi:10.3390/s26216923)
Supplement: Supplementary file 1 [file sensors-26-06923-s001.zip › Supplemental Figure S3.pdf]

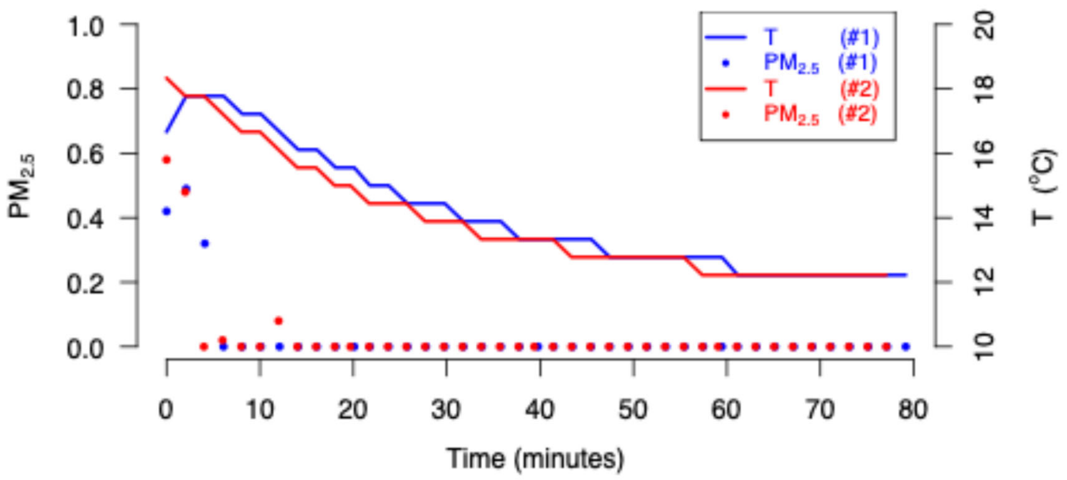

Supplemental Figure S3: Time series of temperature (internal) and $PM_{2.5}$ levels logged by the SD cards of two air monitors (#1 and #2) that were placed at zero time into a 4°C walk-in refrigerator. The time series end at about 80 min when both monitors stopped logging data to the SD card. $PM_{2.5}$ levels quickly approach 0 μg/m^3 in the refrigerator due to lack of air exchange.
